# Stereoacuity and Related Factors: The Shandong Children Eye Study

**DOI:** 10.1371/journal.pone.0157829

**Published:** 2016-07-08

**Authors:** Da-dong Guo, Jian-feng Wu, Yuan-yuan Hu, Wei Sun, Tai-liang Lv, Wen-jun Jiang, Hui Wu, Xing-rong Wang, Jost B. Jonas, Hong-sheng Bi

**Affiliations:** 1 Eye Institute of Shandong University of Traditional Chinese Medicine, Jinan, Shandong, China; 2 Ophthalmology & Optometry School, Shandong University of Traditional Chinese Medicine, Jinan, Shandong, China; 3 The Second Affiliated Hospital of Shandong University of Traditional Chinese Medicine, Jinan, Shandong, China; 4 Department of Ophthalmology, Medical Faculty Mannheim of the Ruprecht-Karls-University Heidelberg, Germany; University of Iowa, UNITED STATES

## Abstract

**Objective:**

To assess stereoacuity in a population-based sample of children and to examine ocular and systemic parameters related to stereoacuity.

**Methods:**

Using a random cluster sampling method, four- to 18-year-old children from kindergartens, elementary schools, junior high schools and senior high schools from a rural area and an urban area in the East Chinese province of Shandong were included in the school-based cross-sectional study. All participants underwent a comprehensive eye examination including assessment of cycloplegic refraction and measurement of stereoacuity using the Titmus Stereo test.

**Results:**

Out of 6364 eligible children, 5780 (90.8%) children with a mean age of 10.1 ± 3.2 years (range: 4 to 18 years) participated. Mean (± standard deviation) stereoacuity was 50.2 ± 50.6 arc seconds. Stereoacuity improved significantly (*P*<0.01) from the age group of 4 years to the age group of 6 to 7 years, then showed a plateau, deteriorated (*P* = 0.001) for both sexes from the age group of 9 years to the age group of 12 years (*P*<0.001), after which it improved (*P* = 0.001) again in the age group of 16 years or older to the pre-puberty values. In multivariate analysis, larger angle of binocular disparity (i.e., lower stereoacuity) was significantly associated with lower best corrected visual acuity (logMAR; *P*<0.001), higher intereye difference in refractive error (spherical equivalent) (*P*<0.001), higher cylindrical refractive error (*P*<0.001), higher refractive error (spherical value; *P*<0.001), higher intereye difference in best corrected visual acuity (logMAR) (*P* = 0.001), higher intereye difference in axial length (*P* = 0.001), and rural region of habitation (*P* = 0.006).

**Conclusions:**

Stereoacuity as tested with the Titmus Stereo test improved significantly from an age of 4 years to an age of 6 and 7 years, then remained constant, temporarily deteriorated for both sexes in pre-puberty and puberty, after which it improved again to pre-puberty or better values at the age of 16 years or older. Lower stereoacuity was associated with lower best corrected visual acuity and higher intereye difference in best corrected visual acuity, higher cylindrical and spherical refractive errors, higher inter-eye difference in refractive error, higher intereye difference in axial length, and rural region of habitation.

## Introduction

Stereopsis is an important quality of normal human vision. It has been defined as the perception of depth and depends on the binocular analysis of the three-dimensional structure of objects. Stereopsis is not equivalent to depth perception, but is the type of depth perception that relies on binocularity. It is obtained on the basis of visual information deriving from binocular vision. Binocular retinal disparity is an essential component of stereopsis [1,2]. Threshold stereoacuity refers to the smallest angle of binocular disparity that can provoke perception of depth or stereopsis. It has been reported that in the normal development, distance stereoacuity reaches adult levels at an age of approximately 5 years [3]. Disruption of normal binocular fusion due to reasons such as amblyopia, anisometropia or aniseikonia decreases stereoacuity and secondarily may have an impact on the development of fine visual-motor actions and spatial resolution [4]. Stereoacuity has commonly been assessed clinically by tests such as the Titmus stereo test [5,6].

Previous studies on children assessed associations between stereoacuity and parameters such as anisometropic amblyopia, myopic ametropia, and anisometropia [5,7–10]. Most of these studies on stereoacuity included a relevant small number of children or they examined only relatively few parameters, so that a multivariate analysis to explore associations between stereoacuity and ocular or systemic factors did not include all parameters of interest. We therefore performed the current school-based study on a relatively large number of children aged 4 to 18years, measured stereoacuity and investigated the associations between stereoacuity and other ocular and systemic parameters in a multivariate manner. We choose a school-based recruitment of study participants coming from different regions of the East Chinese province of Shandong to reduce the potential bias by referral of children as it can be the case in hospital-based studies.

## Methods

Shandong is a coastal province in Eastern China. The school-based cross-sectional Shandong Children Eye Study was conducted in the rural county of Guanxian in Western Shandong and the relatively highly developed city of Weihai in Eastern Shandong. The study was approved by the Ethics Board of the Eye Institute of Shandong University of Traditional Chinese Medicine and the Local Administration of the Education and School Board of Shandong Province. The parents or legal guardians of the children signed an informed consent. All research procedures and data collection were performed in a manner compliant with the related legal requirements, and all research procedures adhered to the tenets of the Declaration of Helsinki. According to the regional level of social and economic development, the study sites in Guanxian and in Weihai were chosen as representative regions of a rural region and an urban region, respectively, of the province of Shandong. Due to the stratification of clusters by grade and age, children of all ages from 4 years to 18 years were representatively included into the study samples. The stratified cluster sampling method and the calculation of the sample size were reported in detail previously [11–13].

All participants in the present study underwent an interview with a standardized questionnaire. The questionnaire included the questions used in the Refractive Error Study in Children (RESC) studies and questions on items such as birth weight [14]. All answers to the questions were confirmed by the parents or guardians.

The ophthalmological examinations included measurement of stereovision and ocular motility, non-contact tonometry (Topcon CT80, Topcon Co. Tokyo, Japan), ocular biometry by laser interferometry (IOL- Master, V5.0, Carl Zeiss Meditec AG, Jena, Germany), assessment of uncorrected visual acuity and best corrected visual acuity, and slit-lamp assisted biomicroscopy of the anterior and posterior segment of the eye. Using an auto-refractor (KR8900, Topcon, Itabashi, Tokyo, Japan), the refractive status was measured before and after inducing cycloplegia. According to the procedures in the manufacturer’s instruction manual, the vertex distance was 12 mm and the measurement step size was 0.25 diopters for the assessment of the spherical power and cylindrical power. Three measurements were carried out and the mean value was recorded as the final measurement. The difference between the maximum and minimum value of the measurements of spherical refractive error and cylindrical refractive error had to be less than 0.5 diopters, otherwise the measurements were repeated. Cycloplegia was induced by instilling 1% cyclopentolate eye drops (Alcon, Ft. Worth, Texas, USA) at least three times into each eye, except for eyes with diseases and except for children with an intraocular pressure higher than 25 mmHg in any eye.

Stereoacuity was assessed using the Titmus stereo test (Stereo Optical Co., Inc., Chicago, IL, USA). The Titmus test possesses disparities ranging from 800 arc seconds to 40 arc seconds in unequal step sizes. Through a pair of polarizing glasses, subjects viewed the stereogram at a distance of 40 cm, and were asked to seize the wings of the fly. If the participant tried to seize the wings of the fly, the participant was asked to point to the circle that seemed to "jump" out of the book. If a mistake occurred, the preceding target was re-assessed. If an accurate response was repeatedly obtained on the preceding target, the target's disparity was regarded as the measurement value. Testing started with the largest disparity, and inability to correctly identify the target with the largest disparity was recorded as ‘nil’ stereo. The stereoacuity examinations were performed by a small group of trained technicians who were instructed to carry out the test under standardized conditions close to a window during daytime.

Using a commercially available statistical software package (SPSS for Windows, version 22.0, IBM-SPSS, Chicago, IL), we first transformed the results of visual acuity measurements into logarithmic values according to the minimal angle of resolution (logMAR), expressed the stereoacuity measurements as logarithmic values, and we presented the distribution of stereoacuity. We formulated the hypothesis that stereoacuity could be associated with age, gender and region of habitation of the children, with parameters of the optical system of the eye, namely the axial length and the spherical and cylindrical refractive error and the combination of both parameters (i.e., the spherical equivalent) of both eyes as a mean and as intereye difference, and with best corrected visual acuity as a mean of both eyes and as intereye difference. In a second step, we then assessed which of these parameters were associated with the degree of stereoacuity (expressed as logarithmic value) in univariate analysis. We applied Bonferroni´s method to correct for performing multiple statistical analyses. In a third step, we carried out a multi variable regression analyses which included stereoacuity (expressed as logarithmic value) as dependent variables and as independent variables all those parameters which were significantly associated with stereoacuity in the univariate analysis. We then dropped step-by-step those parameters which either showed a high collinearity or which were no longer significantly associated with stereoacuity. In a fourth step of the analysis, we re-added the earlier eliminated parameters and checked whether their exclusion was till justified. All *P*-values were calculated at a 2-tailed level and were regarded as statistically significant when the *P*-value was less than 0.05.

## Results

Out of a total of 6364 eligible children, 339 (5.3%) children did not participate in the clinical examinations, mainly to avoid the temporary effects of cycloplegia, and 245 (3.9%) children did not complete the stereoacuity examination or did not give reliable stereoacuity measurements, so that the study eventually included 5780 (90.8%) children (3027 (52.4%) boys). The mean age was 10.1 ± 3.2 years (median: 10.00 years; range: 4 to 18 years), mean axial length was 23.5 ± 1.2 mm (median: 23.4 mm; range: 12.10 mm to 28.59 mm), and the mean refractive error was -0.27 ± 2.09 diopters (median: +0.50 diopter; range: -11.75 diopters to +10.50 diopters) for the right eyes, and -0.18 ± 2.08 diopters (median: 0.50 diopters; range: -11.75 diopters to +11.00 diopters) for the left eyes. The group of eligible children, who were not included into the study, as compared with the group of children participating in the study, was significantly (*P*<0.001) younger (6.3 ± 2.7 years versus 10.1 ± 3.2 years), and due to the correlation between older age and development of axial myopia, it was significantly (*P*<0.001) less myopic (1.41 ± 1.70 diopters versus -0.27 ± 2.09 diopters (right eyes)) and had a shorter axial length (22.6 ± 4.6 mm versus 23.5 ± 1.2 mm).

Mean stereoacuity was (mean ± standard deviation) 50.2 ± 50.6 seconds of arc (median: 40 arc sec; range: 40 arc sec to 800 arc sec) (Table 1). Stratified into good stereoacuity (≤100 arc sec), moderate stereoacuity (101–799 arc sec), and poor or no stereoacuity (≥800 arc sec), 5597 (96.8%) children had good stereoacuity, 170 (2.9%) children had moderate stereoacuity, and 13 (0.2%) children had poor or no stereoacuity (Table 2). The cut-off values for the stratification of stereopsis were chosen arbitrarily.

**Table 1:** Distribution of Stereoacuity in the Shandong Children Eye Study.

Stereoacuity Arc Seconds)	Frequency (n)	Percentage (%)	Cumulative Percent (%)
40	4384	75.8	75.8
50	903	15.6	91.5
60	182	3.1	94.6
80	68	1.2	95.8
100	60	1	96.8
140	53	0.9	97.8
200	67	1.2	98.9
400	50	0.9	99.8
800 or >	13	0.2	100

**Table 2:** Distribution Stereoacuity in the Shandong Children Eye Study Stratified by Age, Gender, and Region of Habitation.

Age(Years)	n	Stereoacuity	≤100 arc sec	101–799 arc sec	800 arc s or >
		(Mean ± SD, arc s)	n	n	n
4	94	57.13 ± 44.13	89	5	0
5	251	48.65 ± 36.95	245	6	0
6	374	46.47 ± 17.00	370	4	0
7	637	46.69 ± 38.39	630	6	1
8	738	48.09 ± 43.52	721	16	1
9	546	49.16 ± 47.75	535	10	1
10	697	53.93 ± 64.09	669	25	3
11	587	58.31 ± 72.36	552	33	2
12	485	63.90 ± 95.67	449	31	5
13	436	47.84 ± 35.70	424	12	0
14	340	46.91 ± 28.34	332	8	0
15	227	44.23 ± 18.28	223	4	0
16	134	43.88 ± 16.86	132	2	0
17	130	52.62 ± 50.16	123	7	0
18	104	45.00 ± 16.78	103	1	0
Total	5780	50.88 ± 52.68	5597	170	13
Region/Gender					
Rural boys	1551	53.29 ± 64.92	1497	47	7
Rural girls	1331	51.59 ± 52.26	1284	45	2
Urban boys	1476	50.22 ± 50.49	1432	41	3
Urban girls	1422	48.27 ± 38.38	1384	37	1
Rural	2882	52.51 ± 59.40	2781	92	9
Urban	2898	49.26 ± 44.96	2816	78	4
Boys	3027	51.79 ± 58.34	2929	88	10
Girls	2753	49.88 ± 45.64	2668	82	3

**Fig 1:**
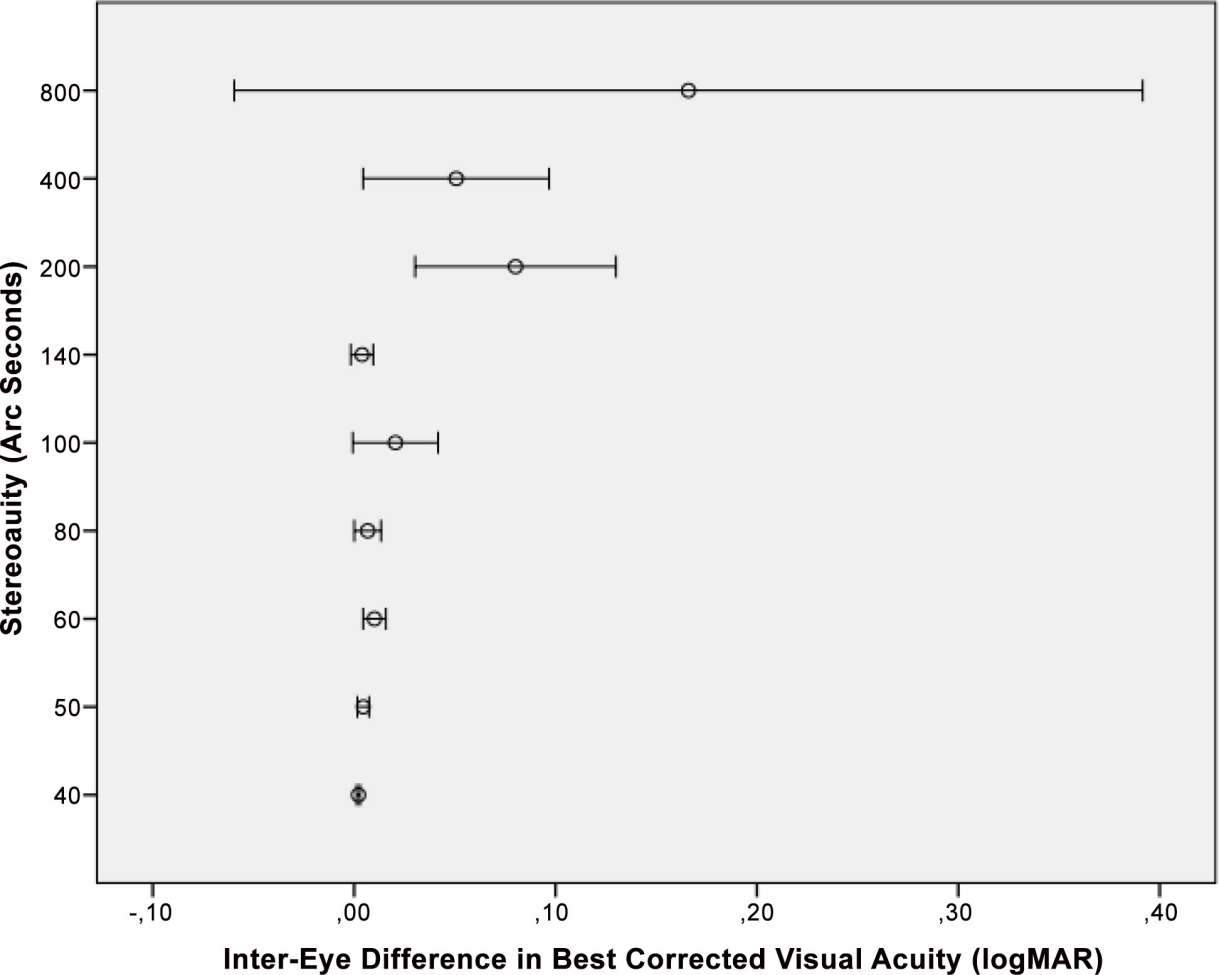
Distribution of Stereoacuity versus the Inter-Eye Difference in Best Corrected Visual Acuity.

**Fig 2:**
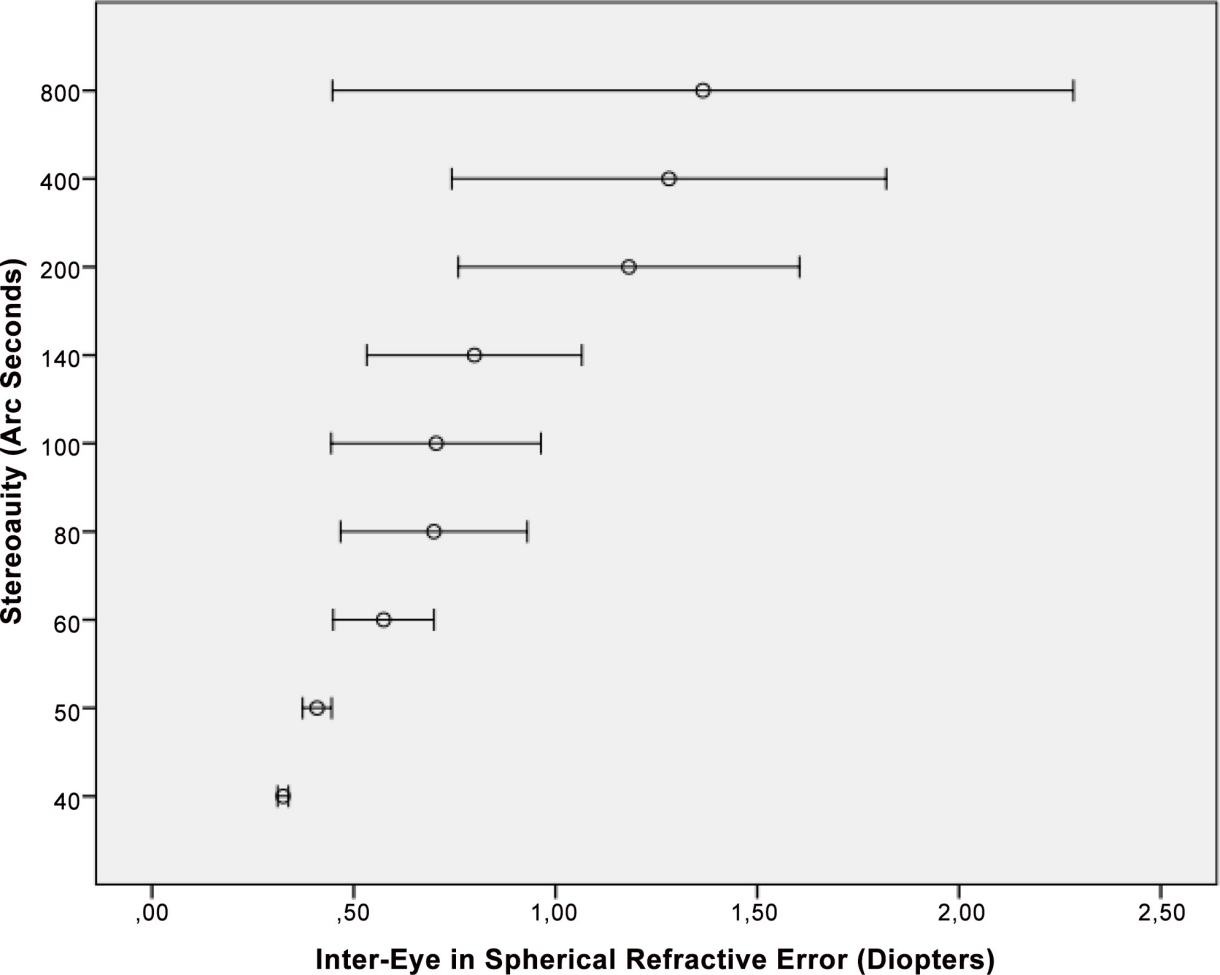
Distribution of Stereoacuity versus the Inter-Eye Difference in Spherical Refractive Error.

**Fig 3:**
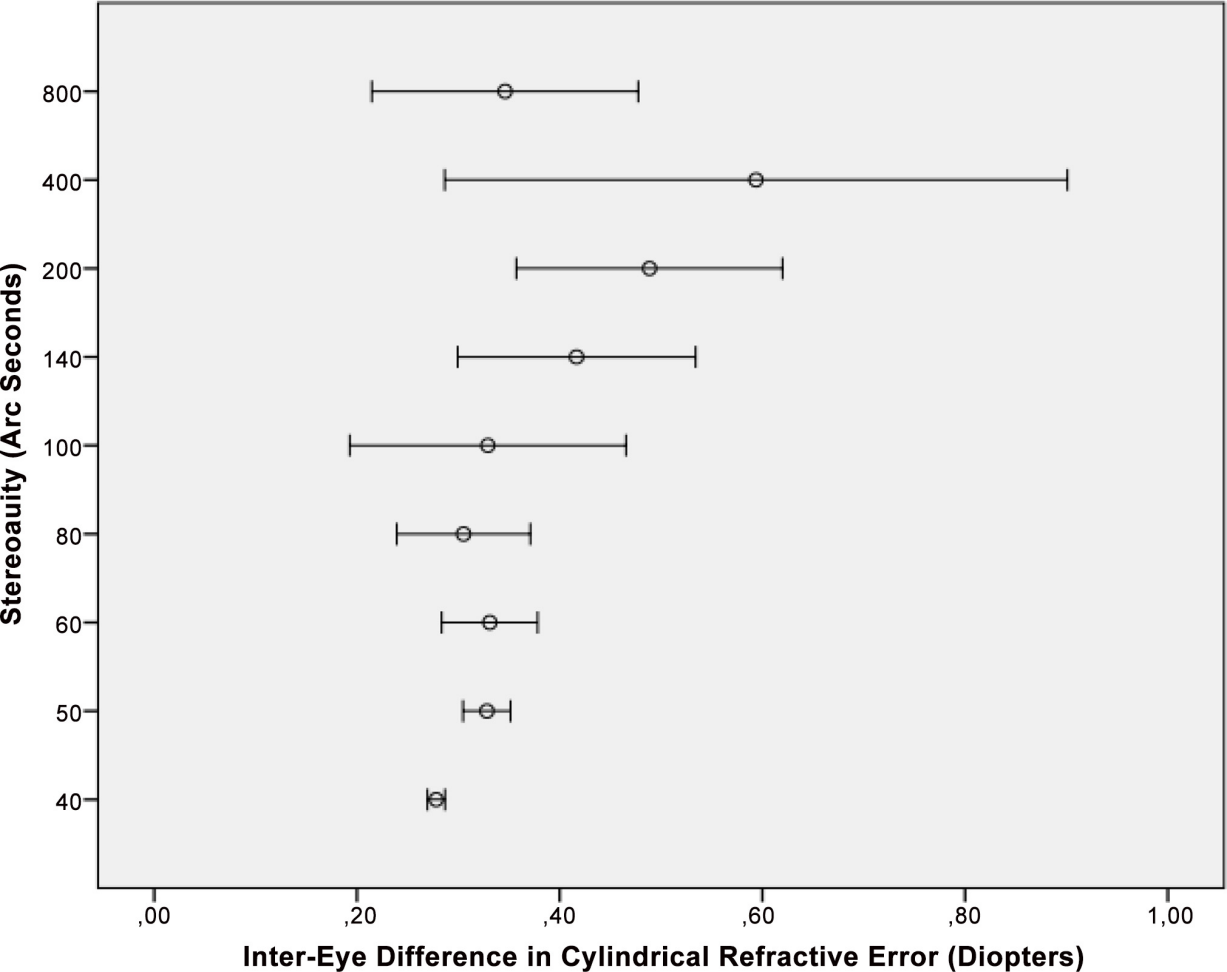
Distribution of Stereoacuity versus the Inter-Eye Difference in Cylindrical Refractive Error.

In univariate analysis (regression analysis), larger angle of binocular disparity (i.e., lower stereoacuity) was significantly associated with lower best corrected visual acuity (i.e., higher LogMAR values) (*P*<0.001), higher interocular difference in best corrected visual acuity (*P*<0.001) (Fig 1), higher interocular difference in spherical refractive error (*P*<0.001) (Fig 2), higher interocular difference in cylindrical refractive error (*P*<0.001) (Fig 3), higher interocular difference in refractive error (spherical equivalent) (*P*<0.001), higher interocular difference in axial length (mm) (*P*<0.001), higher spherical refractive error (diopters) (*P*<0.001), higher cylindrical refractive error (diopters) (*P*<0.001), and rural habitation (*P* = 0.03) (Table 3). Applying Bonferroni´s method for correcting of performing multiple statistical analyses revealed that all significant univariate associations remained significant except for the association between stereoacuity and region of habitation. Stereoacuity was not significantly associated with gender (*P* = 0.13).

**Table 3:** Associations (Univariate Analysis) between Stereoacuity (Expressed as Logarithm of the Measured Angle of Binocular Disparity) and Systemic and Ocular Parameters in the Shandong Children Eye Study.

Parameters	*P*-Value	Standardized Correlation Coefficient
Best Corrected Visual Acuity (LogMAR)	<0.001	0.20
Interocular Difference in Best Corrected Visual Acuity (logMAR)	<0.001	0.22
Interocular Difference in Spherical Refractive Error	<0.001	0.25
Interocular Difference in Cylindrical Refractive Error	<0.001	0.11
Interocular Difference in Refractive Error (Spherical Equivalent)	<0.001	0.24
Interocular Difference in Axial Length (mm)	<0.001	0.20
Spherical refractive Error (Diopters)	<0.001	0.07
Cylindrical Refractive Error (Expressed as–Diopters)	<0.001	-0.12
Rural Versus Urban Region of Habitation	0.03	-0.03
Gender	0.13	
Age	0.68	

**Fig 4:**
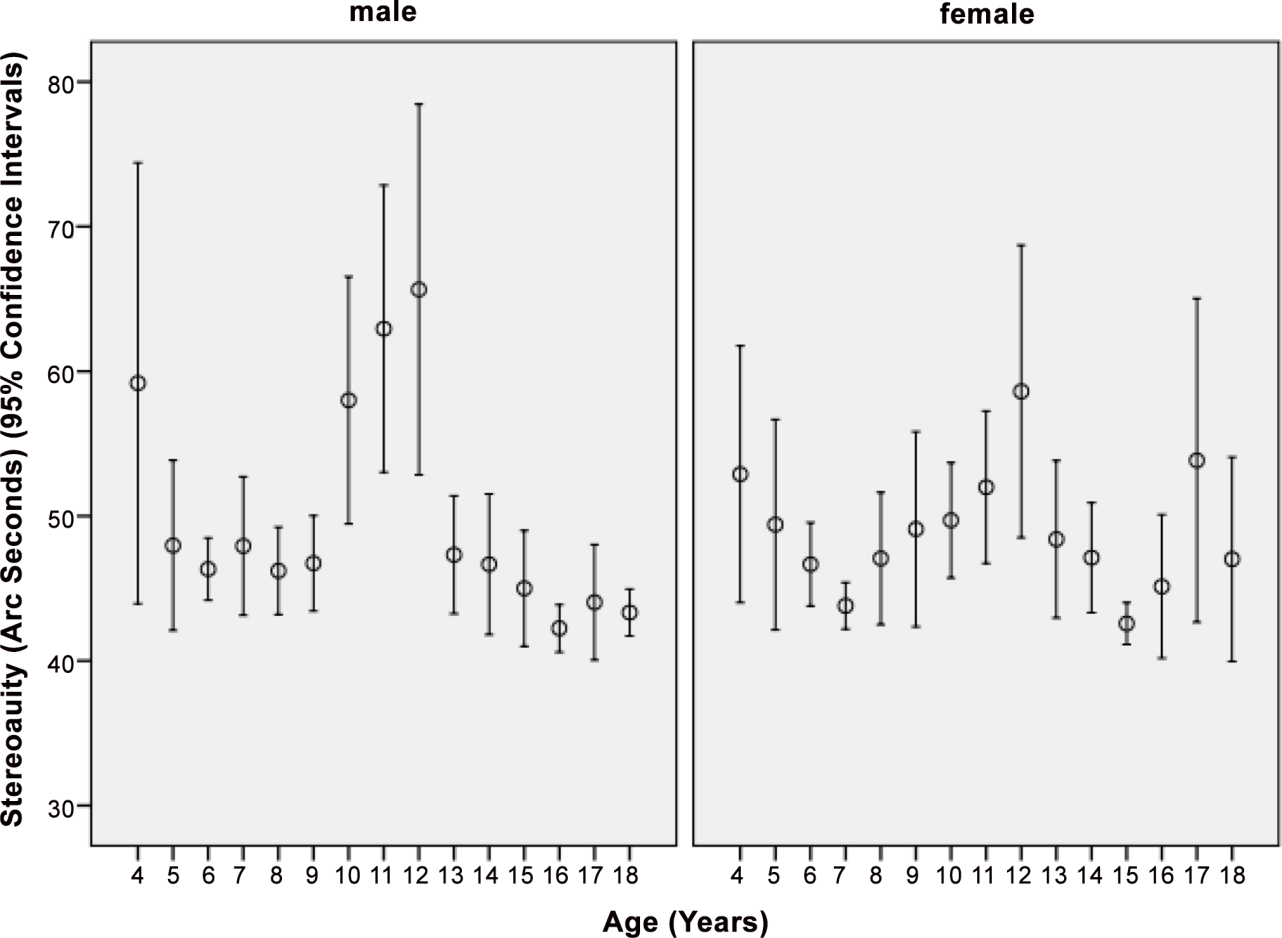
Distribution of Stereoacuity Measured as Minimal Angle of Binocular Disparity in the Shandong Children Eye Study.

Plotting stereoacuity versus age revealed a significant decrease in the angle of binocular disparity (i.e., increasing stereoacuity) in boys from the age group of 4 years to the age group of 6 years (*P* = 0.006; correlation coefficient r: -0.14) and in girls a significant decrease from the age group of 4 years to the age group of 7 years (*P* = 0.001; r: -0.14). It then showed a significant (*P*<0.001) increase in the angle of binocular disparity for both sexes from the age group of 9 years to the age group of 12 years (*P* = 0.001; r: 0.07), after which to the age group of 16 years or older, stereopsis measured as the angle of binocular disparity significantly decreased to the pre-puberty values (*P* = 0.001; r: -0.08) (Fig 4). These associations remained to be statistical significant after application of Bonferroni´s method. Taking the whole study population, stereoacuity was not significantly associated with age (P = 0.68).

In the multivariate analysis, we included all parameters as independent variables which were associated (defined as *P*-value <0.10) with stereoacuity in the univariate analysis. Due to collinearity, we first dropped interocular difference in spherical refractive error (variance inflation factor VIF: 8.65) from the list of independent parameters. We then dropped the intereye difference in cylindrical refractive error (*P* = 0.36) and gender (*P* = 0.10). In the final model (overall correlation coefficient r: 0.32), larger angle of binocular disparity (i.e., lower stereoacuity) was significantly associated with lower best corrected visual acuity (logMAR: *P*<0.001), higher intereye difference in refractive error (spherical equivalent) (*P*<0.001), higher cylindrical refractive error (*P*<0.001), higher refractive error (spherical value; *P*<0.001), higher intereye difference in best corrected visual acuity (logMAR) (*P* = 0.001), higher intereye difference in axial length (*P* = 0.001), rural region of habitation (*P* = 0.006) (Table 4). Also after applying Bonferroni´s method, all these parameters remained to be significantly associated with stereoacuity. We then re-introduced step-by-step the formerly excluded parameters into the model. It showed that gender (*P* = 0.10) and intereye difference in cylindrical refractive error (*P* = 0.36) were again not significantly associated with stereoacuity, and that the interocular difference in spherical refractive error had to be exclude due to high collinearity (VIF: 8.30).

**Table 4:** Associations (Multivariate Analysis) between Stereoacuity (Expressed as Logarithm of the Measured Angle of Binocular Disparity) and Systemic and Ocular Parameters in the Shandong Children Eye Study.

Parameters	*P*-Value	Standardized Correlation Coefficient Beta	Unstandardized Correlation Coefficient B	95% Confidence Interval of B		Variance Inflation Factor
Best Corrected Visual Acuity (logMAR)	<0.001	0.16	0.66	0.51	0.81	2.07
Intereye Difference in Refractive Error (Spherical Equivalent)	<0.001	0.13	0.04	0.03	0.05	1.77
Cylindrical Refractive Error (Expressed as–Diopters)	<0.001	-0.06	-0.02	-0.03	-0.01	1.11
Refractive Error (Spherical Value)	<0.001	0.05	0.004	0.002	0.006	1.05
Intereye Difference in Best Corrected Visual Acuity (logMAR)	0.007	0.05	0.16	0.04	0.27	2.11
Intereye Difference in Axial Length (mm)	0.001	0.05	0.03	0.01	0.05	1.70
Region of Habitation (Rural Versus Urban)	0.006	-0.04	-0.01	-0.02,	-0.003	1.02

## Discussion

In our school-based study on children and teenagers in rural and urban Shandong province in Eastern China, a lower stereoacuity was significantly associated with lower best corrected visual acuity and higher intereye difference in best corrected visual acuity, higher cylindrical and spherical refractive errors, higher inter-eye difference in refractive error, higher intereye difference in axial length, and rural region of habitation. Stereoacuity improved significantly in age group of 4 years to the age group of 6 (boys) to 7 (girls) years, then remained constant, deteriorated for both sexes from the age group of 9 years to the age group of 12 years, after which it improved again to reach pre-puberty or better values at the age of 16 years and older (Fig 4).

The maturation of stereopsis was already examined in previous investigations [15–22]. Simons examined stereopsis in 3- to 5-year-old children and in adults and found that binocular visual development was not complete at 5 years of age [16]. Fox and colleagues determined stereoacuity in children of an age of 3 to 5 years and used a laboratory test combined with procedures designed to optimize the limited attentional, motivational, and response capabilities of young children [17]. They found that the development of stereopsis was not unusually protracted relative to the development of other visual capacities and the authors concurred, that children did not possess the sophisticated cognitive strategies that adults could employ when thresholds were approached and uncertainty was high. The authors concluded that the maturation of the stereoscopic capacity was nearly complete in children at an age of 3 to 5 years [17]. Using the Randot Preschool Stereoacuity Test, Birch and colleagues examined 4355 normal children with an age of 3 to 18 years and 39 adults [19]. They found that the stereoacuity improved from 100 arcsec at 3 years of age to 60 arcsec by 5 years and 40 arcsec by 7 years. A further improvement in stereoacuity was noted beyond 7 years of age to a stereoacuity of 30 arcsec in the 11- to 18-year-old and in the adult groups. They inferred that stereoacuity improved from the age of 3 years to the age of 10 years [19]. Applying the Frisby-Davis distance stereotest, Hong and Park examined 94 visually normal children aged 3 to 11 years and 46 visually normal adults aged 20–49 years [3]. The distance stereoacuity improved significantly (*P*<0.001) from the group of children aged 3 to 5 years (40.6 ± 9.8 arc sec) to the group of children aged 5 to 10 years (14.2 ± 8.2 arc sec), while the latter group of children and the group of adults (12.5 ± 4.8 arc sec) did not differ significantly (*P* = 0.81) in stereoacuity. The authors concluded that distance stereoacuity reached adult levels at approximately 5 years of age [3]. Using the Titmus stereo test, Romano and coworkers examined 321 children with an age of 1.5 to 13 years, who had normal binocular single vision tested. They reported on a gradual improvement in stereoacuity with increasing age up to an age of 9 years when a normal stereoacuity of 40 seconds of arc was found [21]. In the investigation by Oduntan and colleagues, the Randot stereo test was used to measure stereoacuity for 791 male primary school children with an age of 6 to 12 years and with normal vision [22]. The mean stereoacuity decreased from 29.1 arcsec at 6 years to 23.6 arcsec at 11 years. In agreement with the previous studies, which generally found that the maturation of stereopsis was nearly completed at an age 3 to 5 years and that it reached adult levels between 6 to 9 years of age, we also observed in our study that stereopsis improved from 4 to 7 years and that stereopsis almost reached adult levels between 7 to 9 years of age [16–22]. As a new finding, however, we observed a marked deterioration during early puberty, after which stereopsis improved again to finally reach adult levels (Fig 4). This phenomenon was detected for both sexes. One may postulate that the deterioration in stereopsis may be part of the hormonal, physical and psychological transformation which generally takes place in puberty and which is associated with structural brain changes in subcortical brain regions [23–26]. In previous studies, brain changes occurring in puberty also included changes in white matter anisotropy in brain regions like prefrontal regions, the internal capsule, basal ganglia and thalamic pathways, the ventral visual pathways, and the corpus callosum [24]. These brain regions are important for attention, motor skills, cognitive ability, and memory.

The association between worse stereopsis and rural region of habitation in the multivariate analysis may be due to a less advanced development of medical infrastructure, since a higher density of pediatricians may lead to an earlier detection of anisometropia and strabismus, one of the leading causes of poor stereopsis. Although results of stereopsis tests may improve with increased practice and experience with this type of tests, it is unlikely, that the children from the rural region as compared to the children from the urban regions had more test experience, and due to that, better test results.

Better stereopsis was associated with a lower amount of intereye difference in refractive error. It agrees with previous hospital-based investigations by Watanabe and colleagues [27]. Oguz *et al*. observed that an anisometropia of 3 diopters led to a marked reduction in stereoacuity in adult patients [28]. As in our study, previous investigations revealed that decreased visual acuity correlated with reduced stereoacuity [29].

Potential limitations of our study should be mentioned. First, our study had a cross-sectional recruitment of participants so that as in any cross-sectional study in comparison to a longitudinal investigation, we could not examine causal relationships between stereoacuity as the main outcome parameter and other parameters. Second, out of 6364 eligible subjects, 5780 subjects had a complete data set to participate in the present study on stereopsis. As for any population-based study or school-based investigation, non-participation might have led to a bias. The participation rate of 90.8% in our study was relatively high; despite of it however, the group of non-participants and the group of children included into the study differed significantly in age and refractive error. Third, the Titmus Stereo test has monocular clues so that some children may have used these monocular clues to get the correct answers. Fourth, the cooperation of the study participants with the examiners varied and was markedly influenced by the relatively wide range of age of the whole group of study participants. Fifth, the Titmus test as any psychophysical test depends on the concentration of the examinee, so that the relatively poor stereoacuity of the children in puberty could have been caused by a lower degree of concentration of the children. On the other hand, the children in puberty were older than those at an age of 5 to 9 years, and the ability to concentrate on a test usually increases with age. Sixth, although stereoacuity has commonly been assessed clinically by tests such as the Titmus stereo test, one has to acknowledge that the Titmus test does not provide an accurate measure of real stereoacuity, but that it rather pools subjects whose acuities lie within one of 10 different ranges, i.e., 0 to 40, 40 to 50, 50 to 60, 60 to 80, 80 to 100,100 to 140, 140 to 200, 200 to 400, 400 to 800 and 800 or greater min arc [5,6]. Seventh, considering that the technique to measure stereoacuity had a relatively low precision, the testing method might not have been precise enough to detect true small amounts of difference. It may hold true in particular for the relationship between stereoacuity and age (Fig 4). One may also take into account that the stereo test could not measure a stereoacuity of less than 40 arc sec and that approximately 75% of the study participants scored 40 arc sec so that in reality they could have had stereoacuity of better than 40. The finding of a worsening of stereoacuity around the time of early puberty may therefore have to be confirmed in future studies applying tests with a higher precision in testing stereoacuity. Eighth, we analyzed mean values rather than individual values for some of the ophthalmological measurements. If the same number of repetitions are used in all cases this procedure may be acceptable, however a process by which measurements are repeated if the range of initial values exceeds some threshold (e.g., 0.5 diopters) may be liable to understate the variance in the data. This in turn may be likely to overstate the significance of differences and can lead to invalid inferences. It can indicate a potential weakness in statistical analysis.

In conclusion, stereoacuity as tested with the Titmus Stereo test improved significantly from an age of 4 years to an age of 6 to 7 years, then remained constant, deteriorated for both sexes in pre-puberty and puberty, after which it improved again to pre-puberty or better values at the age of 16 years or older. Lower stereoacuity was associated with rural region of habitation, higher cylindrical and spherical refractive error, lower best corrected visual acuity, and higher intereye difference in refractive error.
